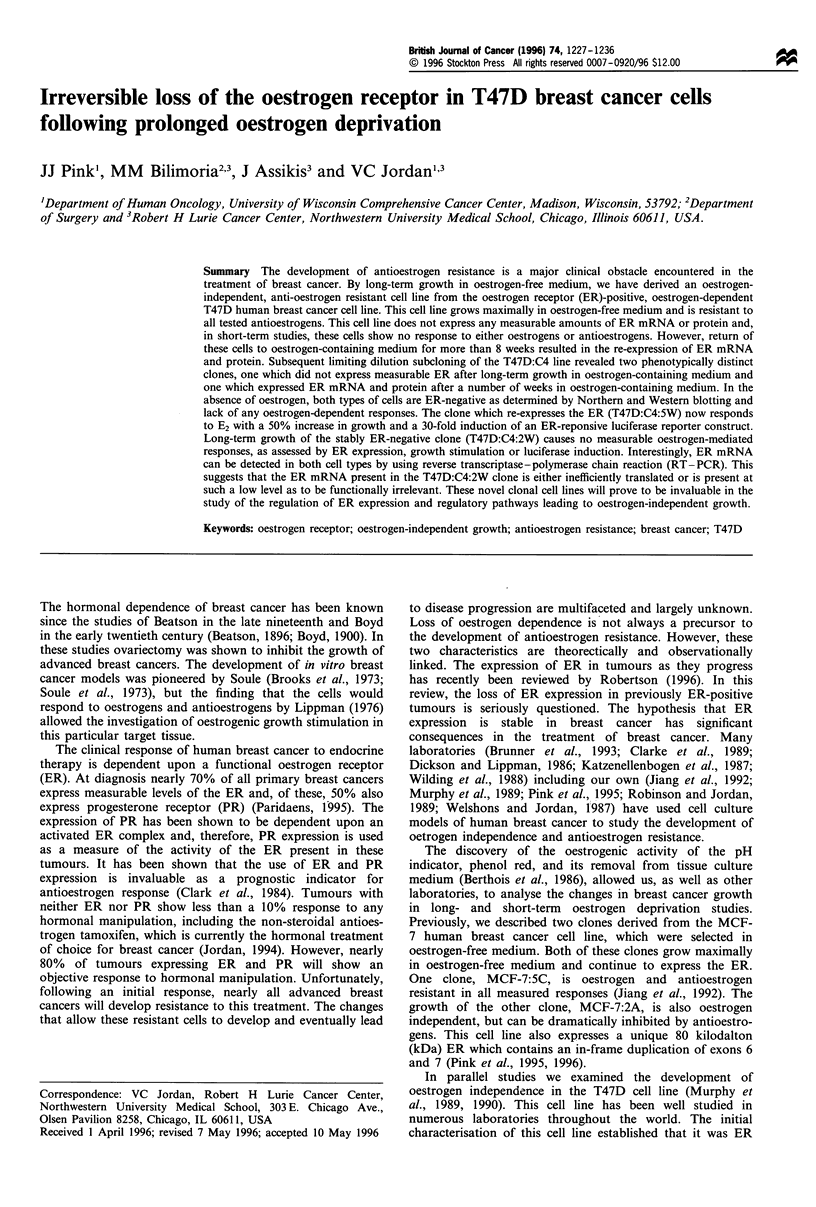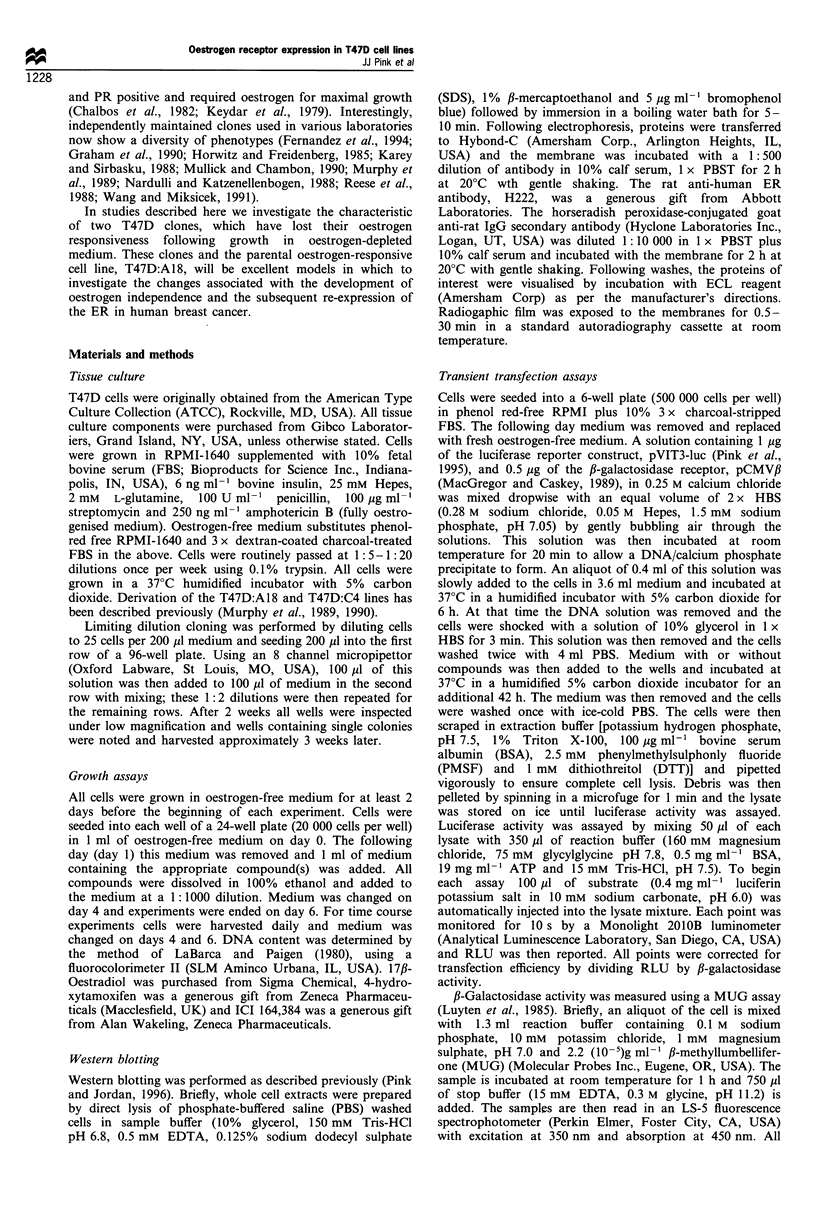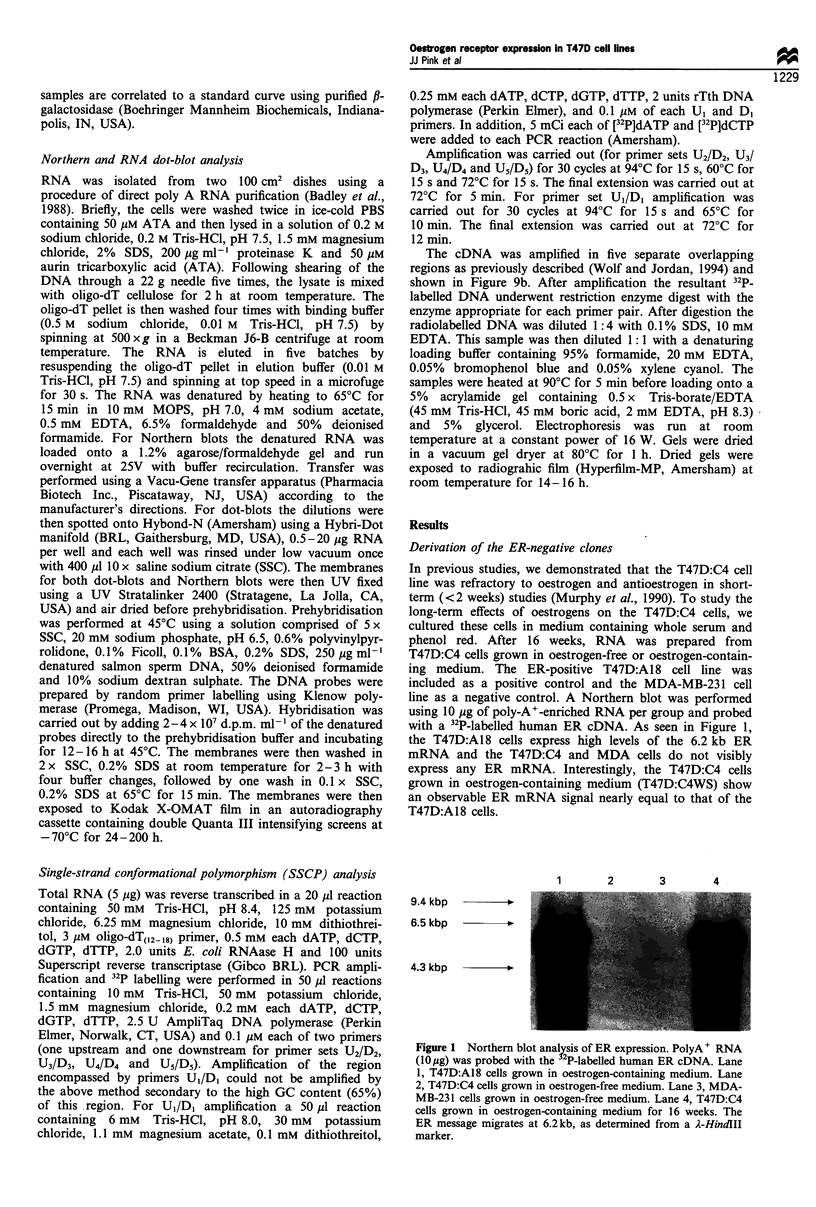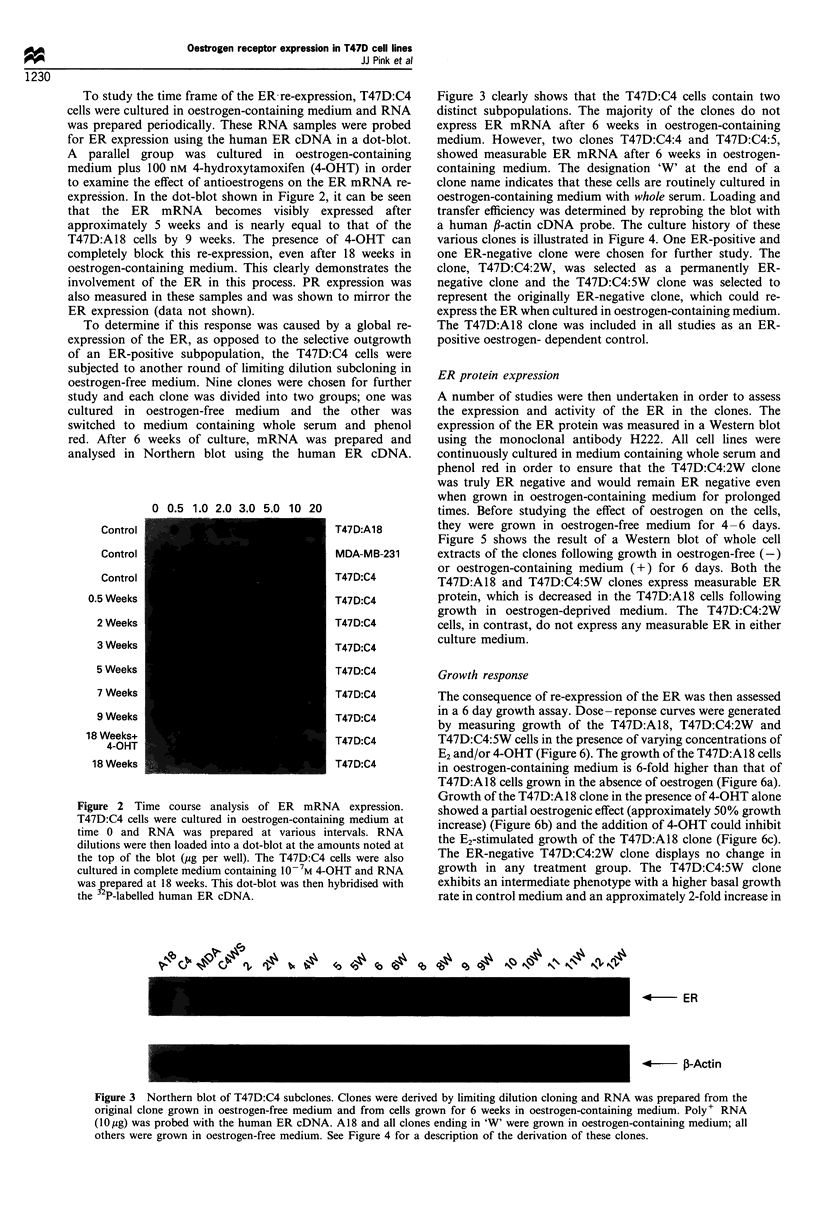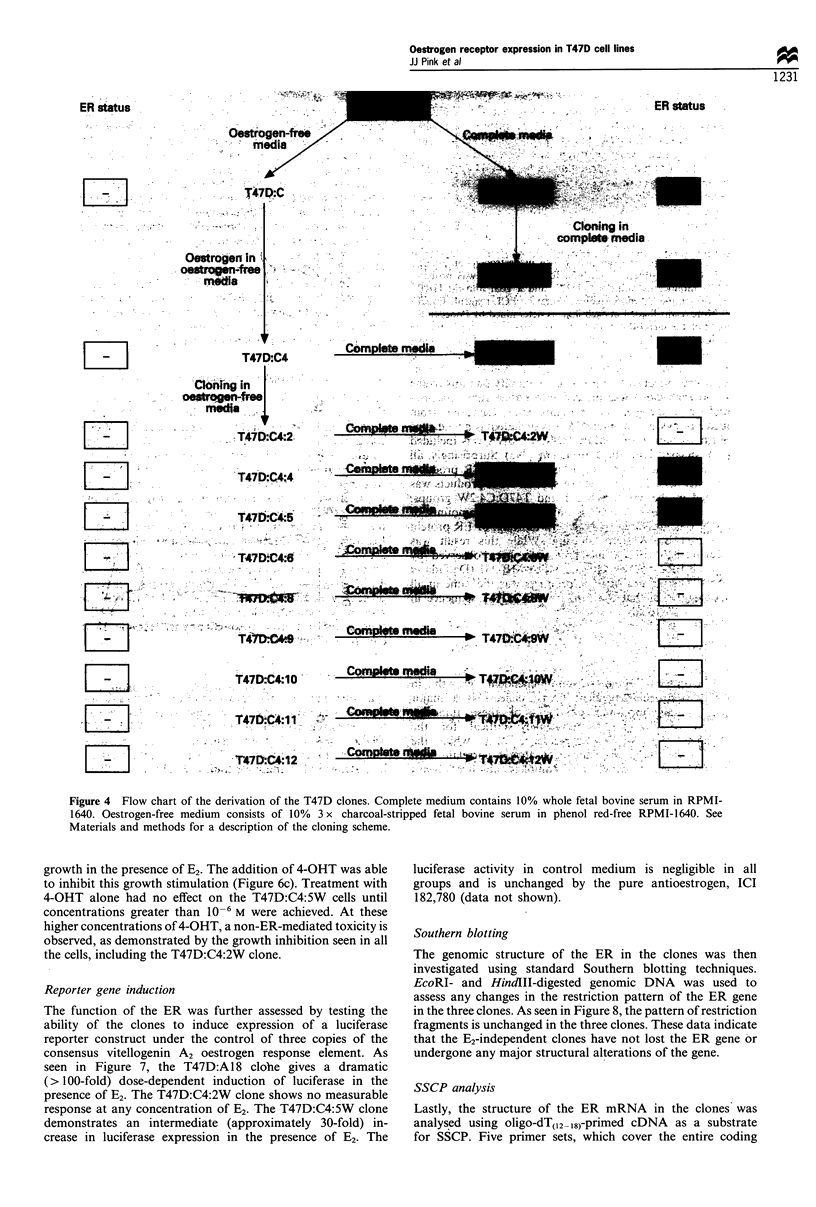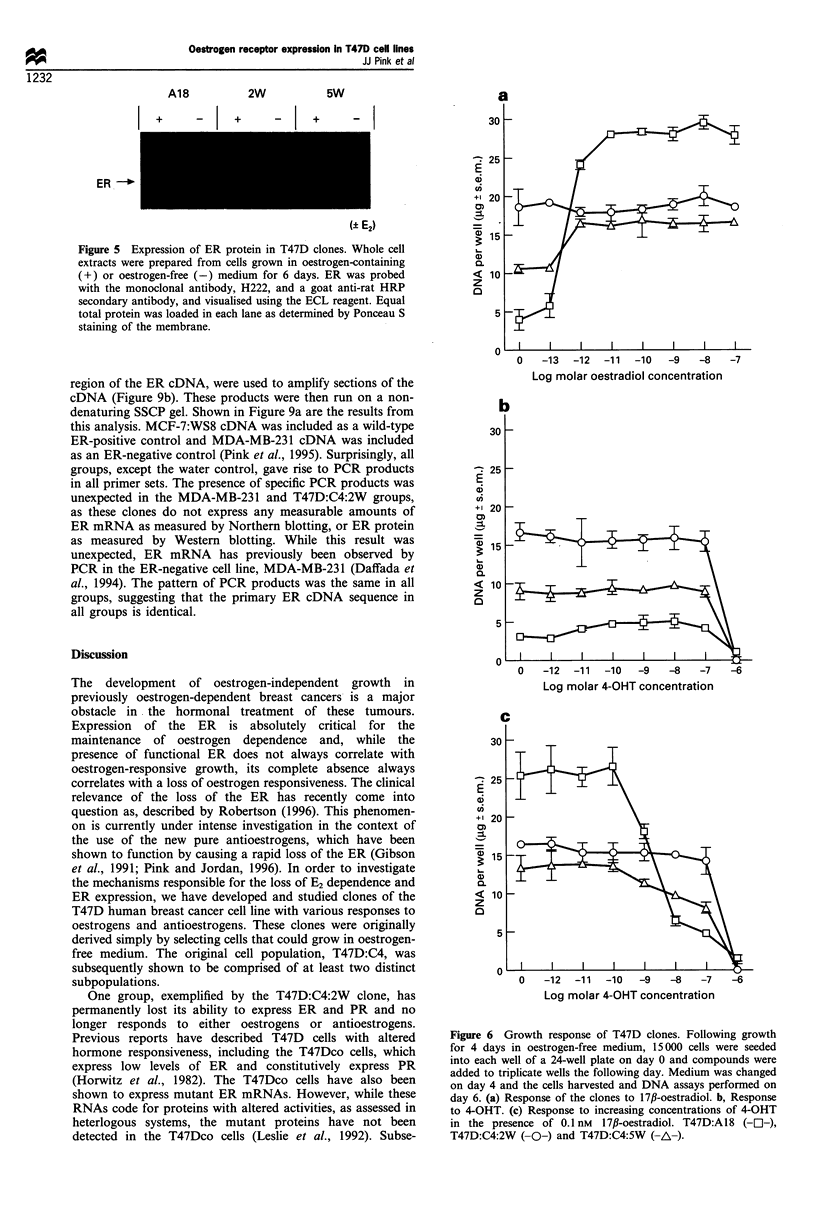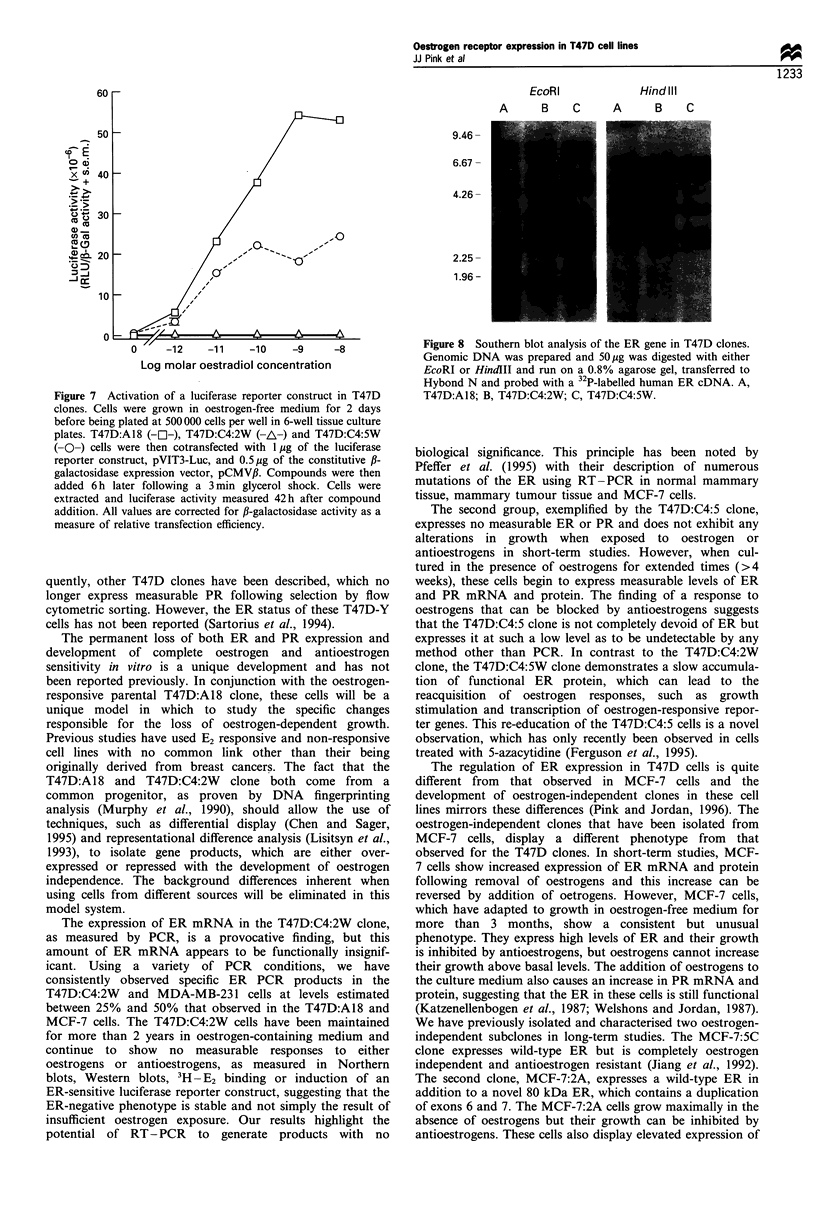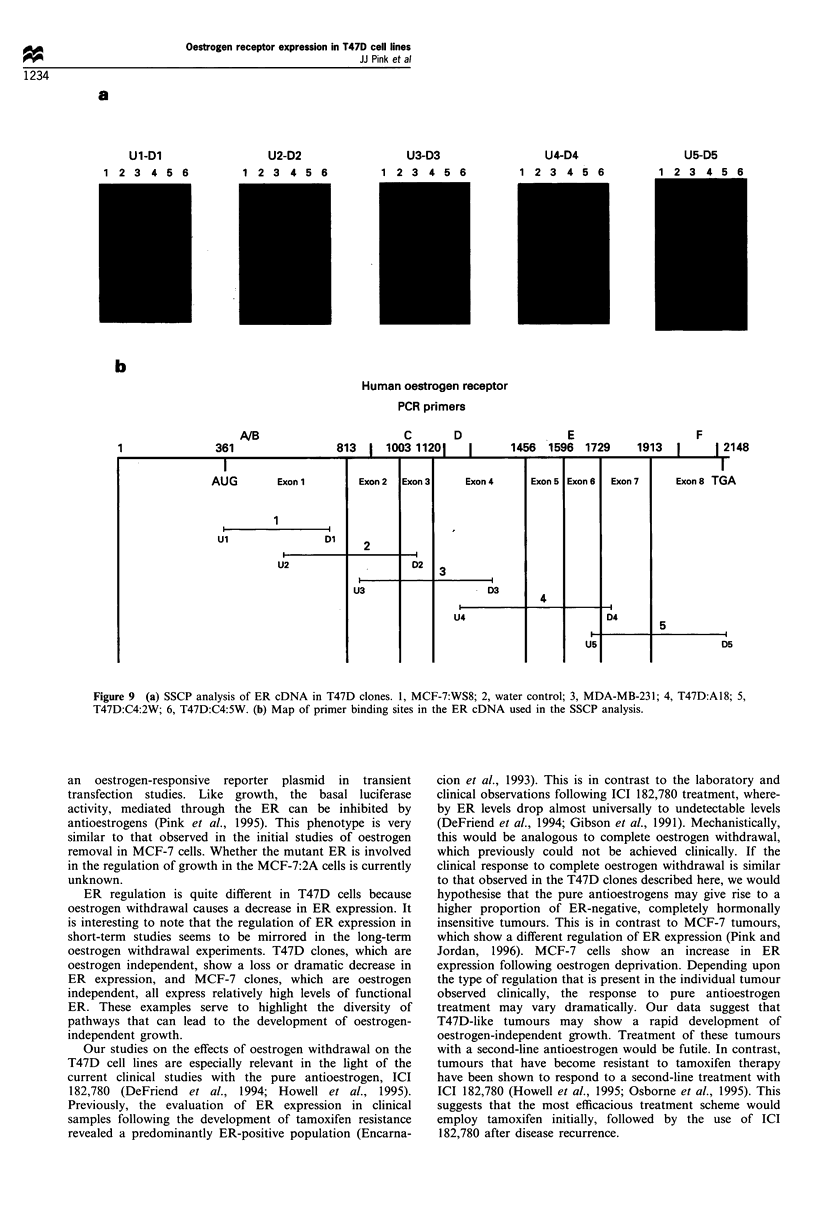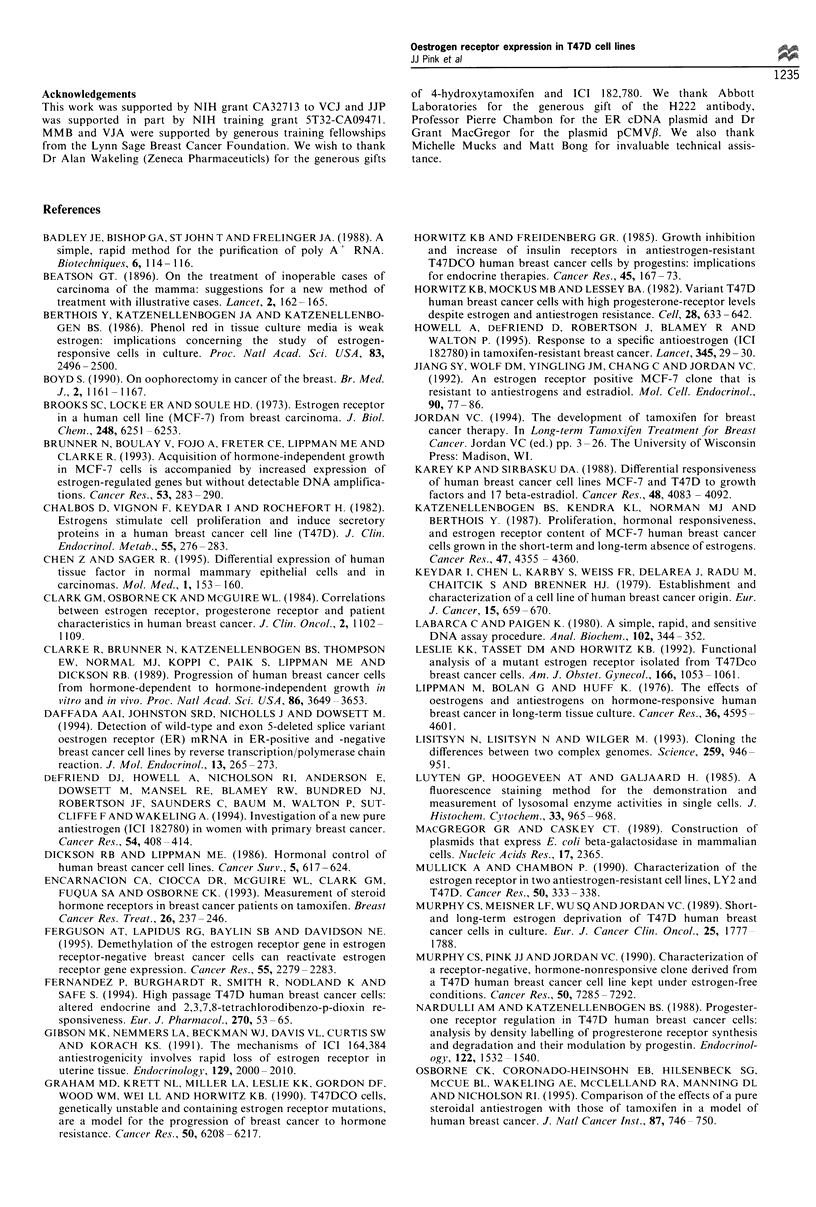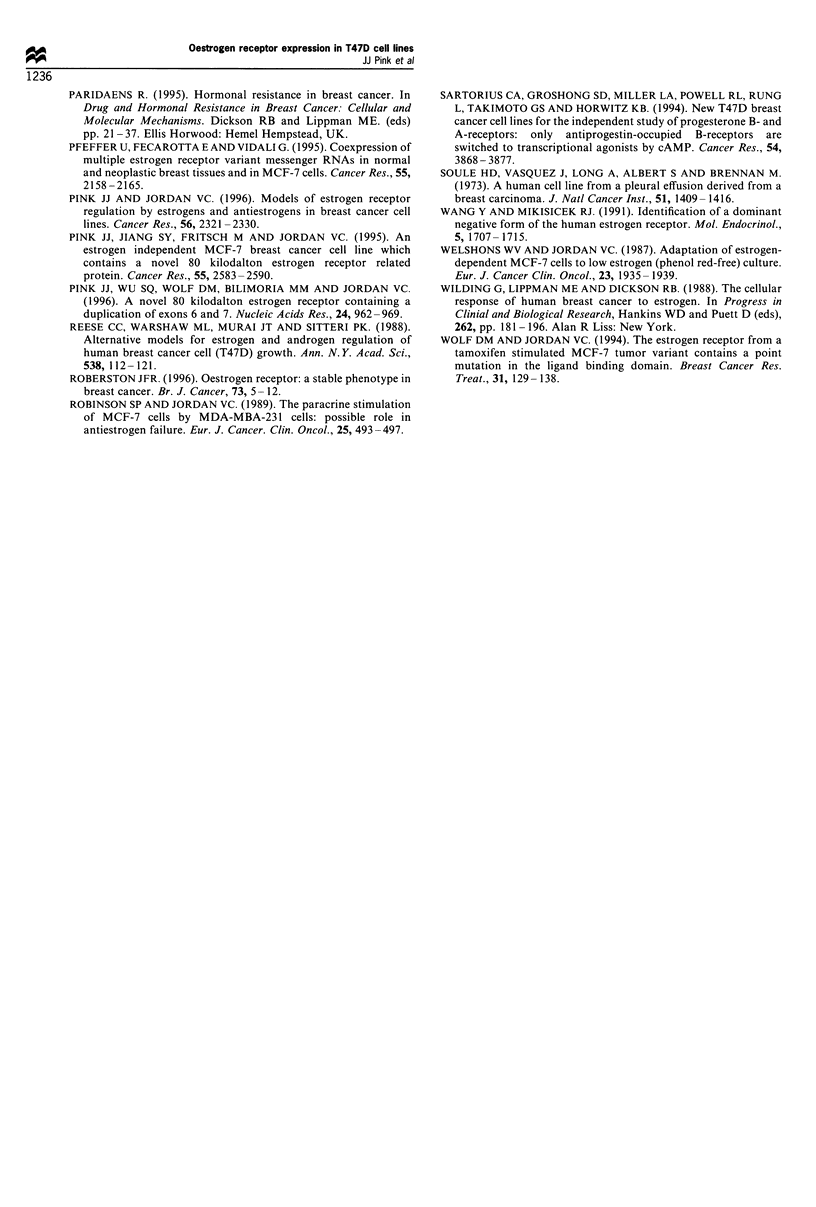# Irreversible loss of the oestrogen receptor in T47D breast cancer cells following prolonged oestrogen deprivation.

**DOI:** 10.1038/bjc.1996.521

**Published:** 1996-10

**Authors:** J. J. Pink, M. M. Bilimoria, J. Assikis, V. C. Jordan

**Affiliations:** Department of Human Oncology, University of Wisconsin Comprehensive Cancer Center, Madison 53792, USA.

## Abstract

**Images:**


British Journal of Cancer (1996) 74, 1227-1236
© 1996 Stockton Press All rights reserved 0007-0920/96 $12.00

### Irreversible loss of the oestrogen receptor in T47D breast cancer cells following prolonged oestrogen deprivation

JJ Pink[1], MM Bilimoria[2,3], J Assikis[3] and VC Jordan[1,3]

[1]*Department of Human Oncology, University of Wisconsin Comprehensive Cancer Center, Madison, Wisconsin, 53792;* [2]*Department of Surgery and* [3]*Robert H Lurie Cancer Center, Northwestern University Medical School, Chicago, Illinois 60611, USA.*

**Summary** The development of antioestrogen resistance is a major clinical obstacle encountered in the treatment of breast cancer. By long-term growth in oestrogen-free medium, we have derived an oestrogen-independent, anti-oestrogen resistant cell line from the oestrogen receptor (ER)-positive, oestrogen-dependent T47D human breast cancer cell line. This cell line grows maximally in oestrogen-free medium and is resistant to all tested antioestrogens. This cell line does not express any measurable amounts of ER mRNA or protein and, in short-term studies, these cells show no response to either oestrogens or antioestrogens. However, return of these cells to oestrogen-containing medium for more than 8 weeks resulted in the re-expression of ER mRNA and protein. Subsequent limiting dilution subcloning of the T47D:C4 line revealed two phenotypically distinct clones, one which did not express measurable ER after long-term growth in oestrogen-containing medium and one which expressed ER mRNA and protein after a number of weeks in oestrogen-containing medium. In the absence of oestrogen, both types of cells are ER-negative as determined by Northern and Western blotting and lack of any oestrogen-dependent responses. The clone which re-expresses the ER (T47D:C4:5W) now responds to $E_2$ with a 50% increase in growth and a 30-fold induction of an ER-reponsive luciferase reporter construct. Long-term growth of the stably ER-negative clone (T47D:C4:2W) causes no measurable oestrogen-mediated responses, as assessed by ER expression, growth stimulation or luciferase induction. Interestingly, ER mRNA can be detected in both cell types by using reverse transcriptase–polymerase chain reaction (RT–PCR). This suggests that the ER mRNA present in the T47D:C4:2W clone is either inefficiently translated or is present at such a low level as to be functionally irrelevant. These novel clonal cell lines will prove to be invaluable in the study of the regulation of ER expression and regulatory pathways leading to oestrogen-independent growth.

**Keywords:** oestrogen receptor; oestrogen-independent growth; antioestrogen resistance; breast cancer; T47D

The hormonal dependence of breast cancer has been known since the studies of Beatson in the late nineteenth and Boyd in the early twentieth century (Beatson, 1896; Boyd, 1900). In these studies ovariectomy was shown to inhibit the growth of advanced breast cancers. The development of *in vitro* breast cancer models was pioneered by Soule (Brooks *et al.*, 1973; Soule *et al.*, 1973), but the finding that the cells would respond to oestrogens and antioestrogens by Lippman (1976) allowed the investigation of oestrogenic growth stimulation in this particular target tissue.

The clinical response of human breast cancer to endocrine therapy is dependent upon a functional oestrogen receptor (ER). At diagnosis nearly 70% of all primary breast cancers express measurable levels of the ER and, of these, 50% also express progesterone receptor (PR) (Paridaens, 1995). The expression of PR has been shown to be dependent upon an activated ER complex and, therefore, PR expression is used as a measure of the activity of the ER present in these tumours. It has been shown that the use of ER and PR expression is invaluable as a prognostic indicator for antioestrogen response (Clark *et al.*, 1984). Tumours with neither ER nor PR show less than a 10% response to any hormonal manipulation, including the non-steroidal antioestrogen tamoxifen, which is currently the hormonal treatment of choice for breast cancer (Jordan, 1994). However, nearly 80% of tumours expressing ER and PR will show an objective response to hormonal manipulation. Unfortunately, following an initial response, nearly all advanced breast cancers will develop resistance to this treatment. The changes that allow these resistant cells to develop and eventually lead to disease progression are multifaceted and largely unknown. Loss of oestrogen dependence is not always a precursor to the development of antioestrogen resistance. However, these two characteristics are theorectically and observationally linked. The expression of ER in tumours as they progress has recently been reviewed by Robertson (1996). In this review, the loss of ER expression in previously ER-positive tumours is seriously questioned. The hypothesis that ER expression is stable in breast cancer has significant consequences in the treatment of breast cancer. Many laboratories (Brunner *et al.*, 1993; Clarke *et al.*, 1989; Dickson and Lippman, 1986; Katzenellenbogen *et al.*, 1987; Wilding *et al.*, 1988) including our own (Jiang *et al.*, 1992; Murphy *et al.*, 1989; Pink *et al.*, 1995; Robinson and Jordan, 1989; Welshons and Jordan, 1987) have used cell culture models of human breast cancer to study the development of oetrogen independence and antioestrogen resistance.

The discovery of the oestrogenic activity of the pH indicator, phenol red, and its removal from tissue culture medium (Berthois *et al.*, 1986), allowed us, as well as other laboratories, to analyse the changes in breast cancer growth in long- and short-term oestrogen deprivation studies. Previously, we described two clones derived from the MCF-7 human breast cancer cell line, which were selected in oestrogen-free medium. Both of these clones grow maximally in oestrogen-free medium and continue to express the ER. One clone, MCF-7:5C, is oestrogen and antioestrogen resistant in all measured responses (Jiang *et al.*, 1992). The growth of the other clone, MCF-7:2A, is also oestrogen independent, but can be dramatically inhibited by antioestrogens. This cell line also expresses a unique 80 kilodalton (kDa) ER which contains an in-frame duplication of exons 6 and 7 (Pink *et al.*, 1995, 1996).

In parallel studies we examined the development of oestrogen independence in the T47D cell line (Murphy *et al.*, 1989, 1990). This cell line has been well studied in numerous laboratories throughout the world. The initial characterisation of this cell line established that it was ER

Correspondence: VC Jordan, Robert H Lurie Cancer Center, Northwestern University Medical School, 303 E. Chicago Ave., Olsen Pavilion 8258, Chicago, IL 60611, USA
Received 1 April 1996; revised 7 May 1996; accepted 10 May 1996

and PR positive and required oestrogen for maximal growth (Chalbos et al., 1982; Keydar et al., 1979). Interestingly, independently maintained clones used in various laboratories now show a diversity of phenotypes (Fernandez et al., 1994; Graham et al., 1990; Horwitz and Freidenberg, 1985; Karey and Sirbasku, 1988; Mullick and Chambon, 1990; Murphy et al., 1989; Nardulli and Katzenellenbogen, 1988; Reese et al., 1988; Wang and Miksicek, 1991).

In studies described here we investigate the characteristic of two T47D clones, which have lost their oestrogen responsiveness following growth in oestrogen-depleted medium. These clones and the parental oestrogen-responsive cell line, T47D:A18, will be excellent models in which to investigate the changes associated with the development of oestrogen independence and the subsequent re-expression of the ER in human breast cancer.

### Materials and methods

### *Tissue culture*

T47D cells were originally obtained from the American Type Culture Collection (ATCC), Rockville, MD, USA. All tissue culture components were purchased from Gibco Laboratoriers, Grand Island, NY, USA, unless otherwise stated. Cells were grown in RPMI-1640 supplemented with 10% fetal bovine serum (FBS; Bioproducts for Science Inc., Indianapolis, IN, USA), 6 ng $ml^{-1}$ bovine insulin, 25 mM Hepes, 2 mM L-glutamine, 100 U $ml^{-1}$ penicillin, 100 $\mu g\ ml^{-1}$ streptomycin and 250 ng $ml^{-1}$ amphotericin B (fully oestrogenised medium). Oestrogen-free medium substitutes phenol-red free RPMI-1640 and 3× dextran-coated charcoal-treated FBS in the above. Cells were routinely passed at 1:5–1:20 dilutions once per week using 0.1% trypsin. All cells were grown in a 37°C humidified incubator with 5% carbon dioxide. Derivation of the T47D:A18 and T47D:C4 lines has been described previously (Murphy et al., 1989, 1990).

Limiting dilution cloning was performed by diluting cells to 25 cells per 200 $\mu l$ medium and seeding 200 $\mu l$ into the first row of a 96-well plate. Using an 8 channel micropipettor (Oxford Labware, St Louis, MO, USA), 100 $\mu l$ of this solution was then added to 100 $\mu l$ of medium in the second row with mixing; these 1:2 dilutions were then repeated for the remaining rows. After 2 weeks all wells were inspected under low magnification and wells containing single colonies were noted and harvested approximately 3 weeks later.

### *Growth assays*

All cells were grown in oestrogen-free medium for at least 2 days before the beginning of each experiment. Cells were seeded into each well of a 24-well plate (20 000 cells per well) in 1 ml of oestrogen-free medium on day 0. The following day (day 1) this medium was removed and 1 ml of medium containing the appropriate compound(s) was added. All compounds were dissolved in 100% ethanol and added to the medium at a 1:1000 dilution. Medium was changed on day 4 and experiments were ended on day 6. For time course experiments cells were harvested daily and medium was changed on days 4 and 6. DNA content was determined by the method of LaBarca and Paigen (1980), using a fluorocolorimeter II (SLM Aminco Urbana, IL, USA). 17β-Oestradiol was purchased from Sigma Chemical, 4-hydroxytamoxifen was a generous gift from Zeneca Pharmaceuticals (Macclesfield, UK) and ICI 164,384 was a generous gift from Alan Wakeling, Zeneca Pharmaceuticals.

### *Western blotting*

Western blotting was performed as described previously (Pink and Jordan, 1996). Briefly, whole cell extracts were prepared by direct lysis of phosphate-buffered saline (PBS) washed cells in sample buffer (10% glycerol, 150 mM Tris-HCl pH 6.8, 0.5 mM EDTA, 0.125% sodium dodecyl sulphate (SDS), 1% β-mercaptoethanol and 5 $\mu g\ ml^{-1}$ bromophenol blue) followed by immersion in a boiling water bath for 5–10 min. Following electrophoresis, proteins were transferred to Hybond-C (Amersham Corp., Arlington Heights, IL, USA) and the membrane was incubated with a 1:500 dilution of antibody in 10% calf serum, 1× PBST for 2 h at 20°C wth gentle shaking. The rat anti-human ER antibody, H222, was a generous gift from Abbott Laboratories. The horseradish peroxidase-conjugated goat anti-rat IgG secondary antibody (Hyclone Laboratories Inc., Logan, UT, USA) was diluted 1:10 000 in 1× PBST plus 10% calf serum and incubated with the membrane for 2 h at 20°C with gentle shaking. Following washes, the proteins of interest were visualised by incubation with ECL reagent (Amersham Corp) as per the manufacturer's directions. Radiogaphic film was exposed to the membranes for 0.5–30 min in a standard autoradiography cassette at room temperature.

### *Transient transfection assays*

Cells were seeded into a 6-well plate (500 000 cells per well) in phenol red-free RPMI plus 10% 3× charcoal-stripped FBS. The following day medium was removed and replaced with fresh oestrogen-free medium. A solution containing 1 $\mu g$ of the luciferase reporter construct, pVIT3-luc (Pink et al., 1995), and 0.5 $\mu g$ of the β-galactosidase receptor, pCMVβ (MacGregor and Caskey, 1989), in 0.25 M calcium chloride was mixed dropwise with an equal volume of 2× HBS (0.28 M sodium chloride, 0.05 M Hepes, 1.5 mM sodium phosphate, pH 7.05) by gently bubbling air through the solutions. This solution was then incubated at room temperature for 20 min to allow a DNA/calcium phosphate precipitate to form. An aliquot of 0.4 ml of this solution was slowly added to the cells in 3.6 ml medium and incubated at 37°C in a humidified incubator with 5% carbon dioxide for 6 h. At that time the DNA solution was removed and the cells were shocked with a solution of 10% glycerol in 1× HBS for 3 min. This solution was then removed and the cells washed twice with 4 ml PBS. Medium with or without compounds was then added to the wells and incubated at 37°C in a humidified 5% carbon dioxide incubator for an additional 42 h. The medium was then removed and the cells were washed once with ice-cold PBS. The cells were then scraped in extraction buffer [potassium hydrogen phosphate, pH 7.5, 1% Triton X-100, 100 $\mu g\ ml^{-1}$ bovine serum albumin (BSA), 2.5 mM phenylmethylsulphonly fluoride (PMSF) and 1 mM dithiothreitol (DTT)] and pipetted vigorously to ensure complete cell lysis. Debris was then pelleted by spinning in a microfuge for 1 min and the lysate was stored on ice until luciferase activity was assayed. Luciferase activity was assayed by mixing 50 $\mu l$ of each lysate with 350 $\mu l$ of reaction buffer (160 mM magnesium chloride, 75 mM glycylglycine pH 7.8, 0.5 mg $ml^{-1}$ BSA, 19 mg $ml^{-1}$ ATP and 15 mM Tris-HCl, pH 7.5). To begin each assay 100 $\mu l$ of substrate (0.4 mg $ml^{-1}$ luciferin potassium salt in 10 mM sodium carbonate, pH 6.0) was automatically injected into the lysate mixture. Each point was monitored for 10 s by a Monolight 2010B luminometer (Analytical Luminescence Laboratory, San Diego, CA, USA) and RLU was then reported. All points were corrected for transfection efficiency by dividing RLU by β-galactosidase activity.

β-Galactosidase activity was measured using a MUG assay (Luyten et al., 1985). Briefly, an aliquot of the cell is mixed with 1.3 ml reaction buffer containing 0.1 M sodium phosphate, 10 mM potassim chloride, 1 mM magnesium sulphate, pH 7.0 and 2.2 ($10^{-5}$)g $ml^{-1}$ β-methyllumbelliferone (MUG) (Molecular Probes Inc., Eugene, OR, USA). The sample is incubated at room temperature for 1 h and 750 $\mu l$ of stop buffer (15 mM EDTA, 0.3 M glycine, pH 11.2) is added. The samples are then read in an LS-5 fluorescence spectrophotometer (Perkin Elmer, Foster City, CA, USA) with excitation at 350 nm and absorption at 450 nm. All samples are correlated to a standard curve using purified β-galactosidase (Boehringer Mannheim Biochemicals, Indianapolis, IN, USA).

### *Northern and RNA dot-blot analysis*

RNA was isolated from two 100 $cm^2$ dishes using a procedure of direct poly A RNA purification (Badley *et al.*, 1988). Briefly, the cells were washed twice in ice-cold PBS containing 50 μM ATA and then lysed in a solution of 0.2 M sodium chloride, 0.2 M Tris-HCl, pH 7.5, 1.5 mM magnesium chloride, 2% SDS, 200 μg $ml^{-1}$ proteinase K and 50 μM aurin tricarboxylic acid (ATA). Following shearing of the DNA through a 22 g needle five times, the lysate is mixed with oligo-dT cellulose for 2 h at room temperature. The oligo-dT pellet is then washed four times with binding buffer (0.5 M sodium chloride, 0.01 M Tris-HCl, pH 7.5) by spinning at 500 × ***g*** in a Beckman J6-B centrifuge at room temperature. The RNA is eluted in five batches by resuspending the oligo-dT pellet in elution buffer (0.01 M Tris-HCl, pH 7.5) and spinning at top speed in a microfuge for 30 s. The RNA was denatured by heating to 65°C for 15 min in 10 mM MOPS, pH 7.0, 4 mM sodium acetate, 0.5 mM EDTA, 6.5% formaldehyde and 50% deionised formamide. For Northern blots the denatured RNA was loaded onto a 1.2% agarose/formaldehyde gel and run overnight at 25V with buffer recirculation. Transfer was performed using a Vacu-Gene transfer apparatus (Pharmacia Biotech Inc., Piscataway, NJ, USA) according to the manufacturer's directions. For dot-blots the dilutions were then spotted onto Hybond-N (Amersham) using a Hybri-Dot manifold (BRL, Gaithersburg, MD, USA), 0.5–20 μg RNA per well and each well was rinsed under low vacuum once with 400 μl 10 × saline sodium citrate (SSC). The membranes for both dot-blots and Northern blots were then UV fixed using a UV Stratalinker 2400 (Stratagene, La Jolla, CA, USA) and air dried before prehybridisation. Prehybridisation was performed at 45°C using a solution comprised of 5 × SSC, 20 mM sodium phosphate, pH 6.5, 0.6% polyvinylpyrrolidone, 0.1% Ficoll, 0.1% BSA, 0.2% SDS, 250 μg $ml^{-1}$ denatured salmon sperm DNA, 50% deionised formamide and 10% sodium dextran sulphate. The DNA probes were prepared by random primer labelling using Klenow polymerase (Promega, Madison, WI, USA). Hybridisation was carried out by adding $2-4 \times 10^7$ d.p.m. $ml^{-1}$ of the denatured probes directly to the prehybridisation buffer and incubating for 12–16 h at 45°C. The membranes were then washed in 2 × SSC, 0.2% SDS at room temperature for 2–3 h with four buffer changes, followed by one wash in 0.1 × SSC, 0.2% SDS at 65°C for 15 min. The membranes were then exposed to Kodak X-OMAT film in an autoradiography cassette containing double Quanta III intensifying screens at −70°C for 24–200 h.

### *Single-strand conformational polymorphism (SSCP) analysis*

Total RNA (5 μg) was reverse transcribed in a 20 μl reaction containing 50 mM Tris-HCl, pH 8.4, 125 mM potassium chloride, 6.25 mM magnesium chloride, 10 mM dithiothreitol, 3 μM oligo-$dT_{(12-18)}$ primer, 0.5 mM each dATP, dCTP, dGTP, dTTP, 2.0 units *E. coli* RNAase H and 100 units Superscript reverse transcriptase (Gibco BRL). PCR amplification and $^{32}P$ labelling were performed in 50 μl reactions containing 10 mM Tris-HCl, 50 mM potassium chloride, 1.5 mM magnesium chloride, 0.2 mM each dATP, dCTP, dGTP, dTTP, 2.5 U AmpliTaq DNA polymerase (Perkin Elmer, Norwalk, CT, USA) and 0.1 μM each of two primers (one upstream and one downstream for primer sets $U_2/D_2$, $U_3/D_3$, $U_4/D_4$ and $U_5/D_5$). Amplification of the region encompassed by primers $U_1/D_1$ could not be amplified by the above method secondary to the high GC content (65%) of this region. For $U_1/D_1$ amplification a 50 μl reaction containing 6 mM Tris-HCl, pH 8.0, 30 mM potassium chloride, 1.1 mM magnesium acetate, 0.1 mM dithiothreitol, 0.25 mM each dATP, dCTP, dGTP, dTTP, 2 units rTth DNA polymerase (Perkin Elmer), and 0.1 μM of each $U_1$ and $D_1$ primers. In addition, 5 mCi each of [$^{32}P$]dATP and [$^{32}P$]dCTP were added to each PCR reaction (Amersham).

Amplification was carried out (for primer sets $U_2/D_2$, $U_3/D_3$, $U_4/D_4$ and $U_5/D_5$) for 30 cycles at 94°C for 15 s, 60°C for 15 s and 72°C for 15 s. The final extension was carried out at 72°C for 5 min. For primer set $U_1/D_1$ amplification was carried out for 30 cycles at 94°C for 15 s and 65°C for 10 min. The final extension was carried out at 72°C for 12 min.

The cDNA was amplified in five separate overlapping regions as previously described (Wolf and Jordan, 1994) and shown in Figure 9b. After amplification the resultant $^{32}P$-labelled DNA underwent restriction enzyme digest with the enzyme appropriate for each primer pair. After digestion the radiolabelled DNA was diluted 1:4 with 0.1% SDS, 10 mM EDTA. This sample was then diluted 1:1 with a denaturing loading buffer containing 95% formamide, 20 mM EDTA, 0.05% bromophenol blue and 0.05% xylene cyanol. The samples were heated at 90°C for 5 min before loading onto a 5% acrylamide gel containing 0.5 × Tris-borate/EDTA (45 mM Tris-HCl, 45 mM boric acid, 2 mM EDTA, pH 8.3) and 5% glycerol. Electrophoresis was run at room temperature at a constant power of 16 W. Gels were dried in a vacuum gel dryer at 80°C for 1 h. Dried gels were exposed to radiograhic film (Hyperfilm-MP, Amersham) at room temperature for 14–16 h.

### Results

### *Derivation of the ER-negative clones*

In previous studies, we demonstrated that the T47D:C4 cell line was refractory to oestrogen and antioestrogen in short-term (<2 weeks) studies (Murphy *et al.*, 1990). To study the long-term effects of oestrogens on the T47D:C4 cells, we cultured these cells in medium containing whole serum and phenol red. After 16 weeks, RNA was prepared from T47D:C4 cells grown in oestrogen-free or oestrogen-containing medium. The ER-positive T47D:A18 cell line was included as a positive control and the MDA-MB-231 cell line as a negative control. A Northern blot was performed using 10 μg of poly-$A^+$-enriched RNA per group and probed with a $^{32}P$-labelled human ER cDNA. As seen in Figure 1, the T47D:A18 cells express high levels of the 6.2 kb ER mRNA and the T47D:C4 and MDA cells do not visibly express any ER mRNA. Interestingly, the T47D:C4 cells grown in oestrogen-containing medium (T47D:C4WS) show an observable ER mRNA signal nearly equal to that of the T47D:A18 cells.

**Figure 1** Northern blot analysis of ER expression. Poly$A^+$ RNA (10 μg) was probed with the $^{32}P$-labelled human ER cDNA. Lane 1, T47D:A18 cells grown in oestrogen-containing medium. Lane 2, T47D:C4 cells grown in oestrogen-free medium. Lane 3, MDA-MB-231 cells grown in oestrogen-free medium. Lane 4, T47D:C4 cells grown in oestrogen-containing medium for 16 weeks. The ER message migrates at 6.2 kb, as determined from a λ-*Hind*III marker.

To study the time frame of the ER re-expression, T47D:C4 cells were cultured in oestrogen-containing medium and RNA was prepared periodically. These RNA samples were probed for ER expression using the human ER cDNA in a dot-blot. A parallel group was cultured in oestrogen-containing medium plus 100 nM 4-hydroxytamoxifen (4-OHT) in order to examine the effect of antioestrogens on the ER mRNA re-expression. In the dot-blot shown in Figure 2, it can be seen that the ER mRNA becomes visibly expressed after approximately 5 weeks and is nearly equal to that of the T47D:A18 cells by 9 weeks. The presence of 4-OHT can completely block this re-expression, even after 18 weeks in oestrogen-containing medium. This clearly demonstrates the involvement of the ER in this process. PR expression was also measured in these samples and was shown to mirror the ER expression (data not shown).

To determine if this response was caused by a global re-expression of the ER, as opposed to the selective outgrowth of an ER-positive subpopulation, the T47D:C4 cells were subjected to another round of limiting dilution subcloning in oestrogen-free medium. Nine clones were chosen for further study and each clone was divided into two groups; one was cultured in oestrogen-free medium and the other was switched to medium containing whole serum and phenol red. After 6 weeks of culture, mRNA was prepared and analysed in Northern blot using the human ER cDNA.

**Figure 2** Time course analysis of ER mRNA expression. T47D:C4 cells were cultured in oestrogen-containing medium at time 0 and RNA was prepared at various intervals. RNA dilutions were then loaded into a dot-blot at the amounts noted at the top of the blot (μg per well). The T47D:C4 cells were also cultured in complete medium containing $10^{-7}$M 4-OHT and RNA was prepared at 18 weeks. This dot-blot was then hybridised with the $^{32}$P-labelled human ER cDNA.

Figure 3 clearly shows that the T47D:C4 cells contain two distinct subpopulations. The majority of the clones do not express ER mRNA after 6 weeks in oestrogen-containing medium. However, two clones T47D:C4:4 and T47D:C4:5, showed measurable ER mRNA after 6 weeks in oestrogen-containing medium. The designation 'W' at the end of a clone name indicates that these cells are routinely cultured in oestrogen-containing medium with *whole* serum. Loading and transfer efficiency was determined by reprobing the blot with a human β-actin cDNA probe. The culture history of these various clones is illustrated in Figure 4. One ER-positive and one ER-negative clone were chosen for further study. The clone, T47D:C4:2W, was selected as a permanently ER-negative clone and the T47D:C4:5W clone was selected to represent the originally ER-negative clone, which could re-express the ER when cultured in oestrogen-containing medium. The T47D:A18 clone was included in all studies as an ER-positive oestrogen- dependent control.

### *ER protein expression*

A number of studies were then undertaken in order to assess the expression and activity of the ER in the clones. The expression of the ER protein was measured in a Western blot using the monoclonal antibody H222. All cell lines were continuously cultured in medium containing whole serum and phenol red in order to ensure that the T47D:C4:2W clone was truly ER negative and would remain ER negative even when grown in oestrogen-containing medium for prolonged times. Before studying the effect of oestrogen on the cells, they were grown in oestrogen-free medium for 4–6 days. Figure 5 shows the result of a Western blot of whole cell extracts of the clones following growth in oestrogen-free (−) or oestrogen-containing medium (+) for 6 days. Both the T47D:A18 and T47D:C4:5W clones express measurable ER protein, which is decreased in the T47D:A18 cells following growth in oestrogen-deprived medium. The T47D:C4:2W cells, in contrast, do not express any measurable ER in either culture medium.

### *Growth response*

The consequence of re-expression of the ER was then assessed in a 6 day growth assay. Dose–reponse curves were generated by measuring growth of the T47D:A18, T47D:C4:2W and T47D:C4:5W cells in the presence of varying concentrations of $E_2$ and/or 4-OHT (Figure 6). The growth of the T47D:A18 cells in oestrogen-containing medium is 6-fold higher than that of T47D:A18 cells grown in the absence of oestrogen (Figure 6a). Growth of the T47D:A18 clone in the presence of 4-OHT alone showed a partial oestrogenic effect (approximately 50% growth increase) (Figure 6b) and the addition of 4-OHT could inhibit the $E_2$-stimulated growth of the T47D:A18 clone (Figure 6c). The ER-negative T47D:C4:2W clone displays no change in growth in any treatment group. The T47D:C4:5W clone exhibits an intermediate phenotype with a higher basal growth rate in control medium and an approximately 2-fold increase in

**Figure 3** Northern blot of T47D:C4 subclones. Clones were derived by limiting dilution cloning and RNA was prepared from the original clone grown in oestrogen-free medium and from cells grown for 6 weeks in oestrogen-containing medium. Poly$^+$ RNA (10 μg) was probed with the human ER cDNA. A18 and all clones ending in 'W' were grown in oestrogen-containing medium; all others were grown in oestrogen-free medium. See Figure 4 for a description of the derivation of these clones.

**Figure 4** Flow chart of the derivation of the T47D clones. Complete medium contains 10% whole fetal bovine serum in RPMI-1640. Oestrogen-free medium consists of 10% 3× charcoal-stripped fetal bovine serum in phenol red-free RPMI-1640. See Materials and methods for a description of the cloning scheme.

growth in the presence of $E_2$. The addition of 4-OHT was able to inhibit this growth stimulation (Figure 6c). Treatment with 4-OHT alone had no effect on the T47D:C4:5W cells until concentrations greater than $10^{-6}$ M were achieved. At these higher concentrations of 4-OHT, a non-ER-mediated toxicity is observed, as demonstrated by the growth inhibition seen in all the cells, including the T47D:C4:2W clone.

*Reporter gene induction*

The function of the ER was further assessed by testing the ability of the clones to induce expression of a luciferase reporter construct under the control of three copies of the consensus vitellogenin $A_2$ oestrogen response element. As seen in Figure 7, the T47D:A18 clone gives a dramatic (>100-fold) dose-dependent induction of luciferase in the presence of $E_2$. The T47D:C4:2W clone shows no measurable response at any concentration of $E_2$. The T47D:C4:5W clone demonstrates an intermediate (approximately 30-fold) increase in luciferase expression in the presence of $E_2$. The luciferase activity in control medium is negligible in all groups and is unchanged by the pure antioestrogen, ICI 182,780 (data not shown).

*Southern blotting*

The genomic structure of the ER in the clones was then investigated using standard Southern blotting techniques. *Eco*RI- and *Hin*dIII-digested genomic DNA was used to assess any changes in the restriction pattern of the ER gene in the three clones. As seen in Figure 8, the pattern of restriction fragments is unchanged in the three clones. These data indicate that the $E_2$-independent clones have not lost the ER gene or undergone any major structural alterations of the gene.

*SSCP analysis*

Lastly, the structure of the ER mRNA in the clones was analysed using oligo-$dT_{(12-18)}$-primed cDNA as a substrate for SSCP. Five primer sets, which cover the entire coding

**Figure 5** Expression of ER protein in T47D clones. Whole cell extracts were prepared from cells grown in oestrogen-containing (+) or oestrogen-free (−) medium for 6 days. ER was probed with the monoclonal antibody, H222, and a goat anti-rat HRP secondary antibody, and visualised using the ECL reagent. Equal total protein was loaded in each lane as determined by Ponceau S staining of the membrane.

region of the ER cDNA, were used to amplify sections of the cDNA (Figure 9b). These products were then run on a non-denaturing SSCP gel. Shown in Figure 9a are the results from this analysis. MCF-7:WS8 cDNA was included as a wild-type ER-positive control and MDA-MB-231 cDNA was included as an ER-negative control (Pink *et al.*, 1995). Surprisingly, all groups, except the water control, gave rise to PCR products in all primer sets. The presence of specific PCR products was unexpected in the MDA-MB-231 and T47D:C4:2W groups, as these clones do not express any measurable amounts of ER mRNA as measured by Northern blotting, or ER protein as measured by Western blotting. While this result was unexpected, ER mRNA has previously been observed by PCR in the ER-negative cell line, MDA-MB-231 (Daffada *et al.*, 1994). The pattern of PCR products was the same in all groups, suggesting that the primary ER cDNA sequence in all groups is identical.

### Discussion

The development of oestrogen-independent growth in previously oestrogen-dependent breast cancers is a major obstacle in the hormonal treatment of these tumours. Expression of the ER is absolutely critical for the maintenance of oestrogen dependence and, while the presence of functional ER does not always correlate with oestrogen-responsive growth, its complete absence always correlates with a loss of oestrogen responsiveness. The clinical relevance of the loss of the ER has recently come into question as, described by Robertson (1996). This phenomenon is currently under intense investigation in the context of the use of the new pure antioestrogens, which have been shown to function by causing a rapid loss of the ER (Gibson *et al.*, 1991; Pink and Jordan, 1996). In order to investigate the mechanisms responsible for the loss of $E_2$ dependence and ER expression, we have developed and studied clones of the T47D human breast cancer cell line with various responses to oestrogens and antioestrogens. These clones were originally derived simply by selecting cells that could grow in oestrogen-free medium. The original cell population, T47D:C4, was subsequently shown to be comprised of at least two distinct subpopulations.

One group, exemplified by the T47D:C4:2W clone, has permanently lost its ability to express ER and PR and no longer responds to either oestrogens or antioestrogens. Previous reports have described T47D cells with altered hormone responsiveness, including the T47Dco cells, which express low levels of ER and constitutively express PR (Horwitz *et al.*, 1982). The T47Dco cells have also been shown to express mutant ER mRNAs. However, while these RNAs code for proteins with altered activities, as assessed in heterlogous systems, the mutant proteins have not been detected in the T47Dco cells (Leslie *et al.*, 1992). Subse-

**Figure 6** Growth response of T47D clones. Following growth for 4 days in oestrogen-free medium, 15 000 cells were seeded into each well of a 24-well plate on day 0 and compounds were added to triplicate wells the following day. Medium was changed on day 4 and the cells harvested and DNA assays performed on day 6. (**a**) Response of the clones to 17$\beta$-oestradiol. **b**, Response to 4-OHT. (**c**) Response to increasing concentrations of 4-OHT in the presence of 0.1 nM 17$\beta$-oestradiol. T47D:A18 (-□-), T47D:C4:2W (-○-) and T47D:C4:5W (-△-).

**Figure 7** Activation of a luciferase reporter construct in T47D clones. Cells were grown in oestrogen-free medium for 2 days before being plated at 500 000 cells per well in 6-well tissue culture plates. T47D:A18 (-□-), T47D:C4:2W (-△-) and T47D:C4:5W (-○-) cells were then cotransfected with 1 $\mu$g of the luciferase reporter construct, pVIT3-Luc, and 0.5 $\mu$g of the constitutive $\beta$-galactosidase expression vector, pCMV$\beta$. Compounds were then added 6 h later following a 3 min glycerol shock. Cells were extracted and luciferase activity measured 42 h after compound addition. All values are corrected for $\beta$-galactosidase activity as a measure of relative transfection efficiency.

**Figure 8** Southern blot analysis of the ER gene in T47D clones. Genomic DNA was prepared and 50 $\mu$g was digested with either *Eco*RI or *Hind*III and run on a 0.8% agarose gel, transferred to Hybond N and probed with a $^{32}$P-labelled human ER cDNA. A, T47D:A18; B, T47D:C4:2W; C, T47D:C4:5W.

quently, other T47D clones have been described, which no longer express measurable PR following selection by flow cytometric sorting. However, the ER status of these T47D-Y cells has not been reported (Sartorius *et al*., 1994).

The permanent loss of both ER and PR expression and development of complete oestrogen and antioestrogen sensitivity *in vitro* is a unique development and has not been reported previously. In conjunction with the oestrogen-responsive parental T47D:A18 clone, these cells will be a unique model in which to study the specific changes responsible for the loss of oestrogen-dependent growth. Previous studies have used $E_2$ responsive and non-responsive cell lines with no common link other than their being originally derived from breast cancers. The fact that the T47D:A18 and T47D:C4:2W clone both come from a common progenitor, as proven by DNA fingerprinting analysis (Murphy *et al*., 1990), should allow the use of techniques, such as differential display (Chen and Sager, 1995) and representational difference analysis (Lisitsyn *et al*., 1993), to isolate gene products, which are either over-expressed or repressed with the development of oestrogen independence. The background differences inherent when using cells from different sources will be eliminated in this model system.

The expression of ER mRNA in the T47D:C4:2W clone, as measured by PCR, is a provocative finding, but this amount of ER mRNA appears to be functionally insignificant. Using a variety of PCR conditions, we have consistently observed specific ER PCR products in the T47D:C4:2W and MDA-MB-231 cells at levels estimated between 25% and 50% that observed in the T47D:A18 and MCF-7 cells. The T47D:C4:2W cells have been maintained for more than 2 years in oestrogen-containing medium and continue to show no measurable responses to either oestrogens or antioestrogens, as measured in Northern blots, Western blots, $^3H-E_2$ binding or induction of an ER-sensitive luciferase reporter construct, suggesting that the ER-negative phenotype is stable and not simply the result of insufficient oestrogen exposure. Our results highlight the potential of RT–PCR to generate products with no biological significance. This principle has been noted by Pfeffer *et al.* (1995) with their description of numerous mutations of the ER using RT–PCR in normal mammary tissue, mammary tumour tissue and MCF-7 cells.

The second group, exemplified by the T47D:C4:5 clone, expresses no measurable ER or PR and does not exhibit any alterations in growth when exposed to oestrogen or antioestrogens in short-term studies. However, when cultured in the presence of oestrogens for extended times (>4 weeks), these cells begin to express measurable levels of ER and PR mRNA and protein. The finding of a response to oestrogens that can be blocked by antioestrogens suggests that the T47D:C4:5 clone is not completely devoid of ER but expresses it at such a low level as to be undetectable by any method other than PCR. In contrast to the T47D:C4:2W clone, the T47D:C4:5W clone demonstrates a slow accumulation of functional ER protein, which can lead to the reacquisition of oestrogen responses, such as growth stimulation and transcription of oestrogen-responsive reporter genes. This re-education of the T47D:C4:5 cells is a novel observation, which has only recently been observed in cells treated with 5-azacytidine (Ferguson *et al*., 1995).

The regulation of ER expression in T47D cells is quite different from that observed in MCF-7 cells and the development of oestrogen-independent clones in these cell lines mirrors these differences (Pink and Jordan, 1996). The oestrogen-independent clones that have been isolated from MCF-7 cells, display a different phenotype from that observed for the T47D clones. In short-term studies, MCF-7 cells show increased expression of ER mRNA and protein following removal of oestrogens and this increase can be reversed by addition of oetrogens. However, MCF-7 cells, which have adapted to growth in oestrogen-free medium for more than 3 months, show a consistent but unusual phenotype. They express high levels of ER and their growth is inhibited by antioestrogens, but oestrogens cannot increase their growth above basal levels. The addition of oestrogens to the culture medium also causes an increase in PR mRNA and protein, suggesting that the ER in these cells is still functional (Katzenellenbogen *et al*., 1987; Welshons and Jordan, 1987). We have previously isolated and characterised two oestrogen-independent subclones in long-term studies. The MCF-7:5C clone expresses wild-type ER but is completely oestrogen independent and antioestrogen resistant (Jiang *et al*., 1992). The second clone, MCF-7:2A, expresses a wild-type ER in addition to a novel 80 kDa ER, which contains a duplication of exons 6 and 7. The MCF-7:2A cells grow maximally in the absence of oestrogens but their growth can be inhibited by antioestrogens. These cells also display elevated expression of

**Figure 9** (**a**) SSCP analysis of ER cDNA in T47D clones. 1, MCF-7:WS8; 2, water control; 3, MDA-MB-231; 4, T47D:A18; 5, T47D:C4:2W; 6, T47D:C4:5W. (**b**) Map of primer binding sites in the ER cDNA used in the SSCP analysis.

an oestrogen-responsive reporter plasmid in transient transfection studies. Like growth, the basal luciferase activity, mediated through the ER can be inhibited by antioestrogens (Pink *et al.*, 1995). This phenotype is very similar to that observed in the initial studies of oestrogen removal in MCF-7 cells. Whether the mutant ER is involved in the regulation of growth in the MCF-7:2A cells is currently unknown.

ER regulation is quite different in T47D cells because oestrogen withdrawal causes a decrease in ER expression. It is interesting to note that the regulation of ER expression in short-term studies seems to be mirrored in the long-term oestrogen withdrawal experiments. T47D clones, which are oestrogen independent, show a loss or dramatic decrease in ER expression, and MCF-7 clones, which are oestrogen independent, all express relatively high levels of functional ER. These examples serve to highlight the diversity of pathways that can lead to the development of oestrogen-independent growth.

Our studies on the effects of oestrogen withdrawal on the T47D cell lines are especially relevant in the light of the current clinical studies with the pure antioestrogen, ICI 182,780 (DeFriend *et al.*, 1994; Howell *et al.*, 1995). Previously, the evaluation of ER expression in clinical samples following the development of tamoxifen resistance revealed a predominantly ER-positive population (Encarnacion *et al.*, 1993). This is in contrast to the laboratory and clinical observations following ICI 182,780 treatment, whereby ER levels drop almost universally to undetectable levels (DeFriend *et al.*, 1994; Gibson *et al.*, 1991). Mechanistically, this would be analogous to complete oestrogen withdrawal, which previously could not be achieved clinically. If the clinical response to complete oestrogen withdrawal is similar to that observed in the T47D clones described here, we would hypothesise that the pure antioestrogens may give rise to a higher proportion of ER-negative, completely hormonally insensitive tumours. This is in contrast to MCF-7 tumours, which show a different regulation of ER expression (Pink and Jordan, 1996). MCF-7 cells show an increase in ER expression following oestrogen deprivation. Depending upon the type of regulation that is present in the individual tumour observed clinically, the response to pure antioestrogen treatment may vary dramatically. Our data suggest that T47D-like tumours may show a rapid development of oestrogen-independent growth. Treatment of these tumours with a second-line antioestrogen would be futile. In contrast, tumours that have become resistant to tamoxifen therapy have been shown to respond to a second-line treatment with ICI 182,780 (Howell *et al.*, 1995; Osborne *et al.*, 1995). This suggests that the most efficacious treatment scheme would employ tamoxifen initially, followed by the use of ICI 182,780 after disease recurrence.

**Acknowledgements**
This work was supported by NIH grant CA32713 to VCJ and JJP was supported in part by NIH training grant 5T32-CA09471. MMB and VJA were supported by generous training fellowships from the Lynn Sage Breast Cancer Foundation. We wish to thank Dr Alan Wakeling (Zeneca Pharmaceuticls) for the generous gifts of 4-hydroxytamoxifen and ICI 182,780. We thank Abbott Laboratories for the generous gift of the H222 antibody, Professor Pierre Chambon for the ER cDNA plasmid and Dr Grant MacGregor for the plasmid pCMV$\beta$. We also thank Michelle Mucks and Matt Bong for invaluable technical assistance.

### References

BADLEY JE, BISHOP GA, ST JOHN T AND FRELINGER JA. (1988). A simple, rapid method for the purification of poly A$^+$ RNA. *Biotechniques*, **6**, 114–116.

BEATSON GT. (1896). On the treatment of inoperable cases of carcinoma of the mamma: suggestions for a new method of treatment with illustrative cases. *Lancet*, **2**, 162–165.

BERTHOIS Y, KATZENELLENBOGEN JA AND KATZENELLENBOGEN BS. (1986). Phenol red in tissue culture media is weak estrogen: implications concerning the study of estrogen-responsive cells in culture. *Proc. Natl Acad. Sci. USA*, **83**, 2496–2500.

BOYD S. (1990). On oophorectomy in cancer of the breast. *Br. Med. J.*, **2**, 1161–1167.

BROOKS SC, LOCKE ER AND SOULE HD. (1973). Estrogen receptor in a human cell line (MCF-7) from breast carcinoma. *J. Biol. Chem.*, **248**, 6251–6253.

BRUNNER N, BOULAY V, FOJO A, FRETER CE, LIPPMAN ME AND CLARKE R. (1993). Acquisition of hormone-independent growth in MCF-7 cells is accompanied by increased expression of estrogen-regulated genes but without detectable DNA amplifications. *Cancer Res.*, **53**, 283–290.

CHALBOS D, VIGNON F, KEYDAR I AND ROCHEFORT H. (1982). Estrogens stimulate cell proliferation and induce secretory proteins in a human breast cancer cell line (T47D). *J. Clin. Endocrinol. Metab.*, **55**, 276–283.

CHEN Z AND SAGER R. (1995). Differential expression of human tissue factor in normal mammary epithelial cells and in carcinomas. *Mol. Med.*, **1**, 153–160.

CLARK GM, OSBORNE CK AND MCGUIRE WL. (1984). Correlations between estrogen receptor, progesterone receptor and patient characteristics in human breast cancer. *J. Clin. Oncol.*, **2**, 1102–1109.

CLARKE R, BRUNNER N, KATZENELLENBOGEN BS, THOMPSON EW, NORMAL MJ, KOPPI C, PAIK S, LIPPMAN ME AND DICKSON RB. (1989). Progression of human breast cancer cells from hormone-dependent to hormone-independent growth *in vitro* and *in vivo*. *Proc. Natl Acad. Sci. USA*, **86**, 3649–3653.

DAFFADA AAI, JOHNSTON SRD, NICHOLLS J AND DOWSETT M. (1994). Detection of wild-type and exon 5-deleted splice variant oestrogen receptor (ER) mRNA in ER-positive and -negative breast cancer cell lines by reverse transcription/polymerase chain reaction. *J. Mol. Endocrinol.*, **13**, 265–273.

DEFRIEND DJ, HOWELL A, NICHOLSON RI, ANDERSON E, DOWSETT M, MANSEL RE, BLAMEY RW, BUNDRED NJ, ROBERTSON JF, SAUNDERS C, BAUM M, WALTON P, SUTCLIFFE F AND WAKELING A. (1994). Investigation of a new pure antiestrogen (ICI 182780) in women with primary breast cancer. *Cancer Res.*, **54**, 408–414.

DICKSON RB AND LIPPMAN ME. (1986). Hormonal control of human breast cancer cell lines. *Cancer Surv.*, **5**, 617–624.

ENCARNACION CA, CIOCCA DR, MCGUIRE WL, CLARK GM, FUQUA SA AND OSBORNE CK. (1993). Measurement of steroid hormone receptors in breast cancer patients on tamoxifen. *Breast Cancer Res. Treat.*, **26**, 237–246.

FERGUSON AT, LAPIDUS RG, BAYLIN SB AND DAVIDSON NE. (1995). Demethylation of the estrogen receptor gene in estrogen receptor-negative breast cancer cells can reactivate estrogen receptor gene expression. *Cancer Res.*, **55**, 2279–2283.

FERNANDEZ P, BURGHARDT R, SMITH R, NODLAND K AND SAFE S. (1994). High passage T47D human breast cancer cells: altered endocrine and 2,3,7,8-tetrachlorodibenzo-p-dioxin responsiveness. *Eur. J. Pharmacol.*, **270**, 53–65.

GIBSON MK, NEMMERS LA, BECKMAN WJ, DAVIS VL, CURTIS SW AND KORACH KS. (1991). The mechanisms of ICI 164,384 antiestrogenicity involves rapid loss of estrogen receptor in uterine tissue. *Endocrinology*, **129**, 2000–2010.

GRAHAM MD, KRETT NL, MILLER LA, LESLIE KK, GORDON DF, WOOD WM, WEI LL AND HORWITZ KB. (1990). T47DCO cells, genetically unstable and containing estrogen receptor mutations, are a model for the progression of breast cancer to hormone resistance. *Cancer Res.*, **50**, 6208–6217.

HORWITZ KB AND FREIDENBERG GR. (1985). Growth inhibition and increase of insulin receptors in antiestrogen-resistant T47DCO human breast cancer cells by progestins: implications for endocrine therapies. *Cancer Res.*, **45**, 167–73.

HORWITZ KB, MOCKUS MB AND LESSEY BA. (1982). Variant T47D human breast cancer cells with high progesterone-receptor levels despite estrogen and antiestrogen resistance. *Cell*, **28**, 633–642.

HOWELL A, DEFRIEND D, ROBERTSON J, BLAMEY R AND WALTON P. (1995). Response to a specific antioestrogen (ICI 182780) in tamoxifen-resistant breast cancer. *Lancet*, **345**, 29–30.

JIANG SY, WOLF DM, YINGLING JM, CHANG C AND JORDAN VC. (1992). An estrogen receptor positive MCF-7 clone that is resistant to antiestrogens and estradiol. *Mol. Cell. Endocrinol.*, **90**, 77–86.

JORDAN VC. (1994). The development of tamoxifen for breast cancer therapy. In *Long-term Tamoxifen Treatment for Breast Cancer*. Jordan VC (ed.) pp. 3–26. The University of Wisconsin Press: Madison, WI.

KAREY KP AND SIRBASKU DA. (1988). Differential responsiveness of human breast cancer cell lines MCF-7 and T47D to growth factors and 17 beta-estradiol. *Cancer Res.*, **48**, 4083 – 4092.

KATZENELLENBOGEN BS, KENDRA KL, NORMAN MJ AND BERTHOIS Y. (1987). Proliferation, hormonal responsiveness, and estrogen receptor content of MCF-7 human breast cancer cells grown in the short-term and long-term absence of estrogens. *Cancer Res.*, **47**, 4355 – 4360.

KEYDAR I, CHEN L, KARBY S, WEISS FR, DELAREA J, RADU M, CHAITCIK S AND BRENNER HJ. (1979). Establishment and characterization of a cell line of human breast cancer origin. *Eur. J. Cancer*, **15**, 659–670.

LABARCA C AND PAIGEN K. (1980). A simple, rapid, and sensitive DNA assay procedure. *Anal. Biochem.*, **102**, 344–352.

LESLIE KK, TASSET DM AND HORWITZ KB. (1992). Functional analysis of a mutant estrogen receptor isolated from T47Dco breast cancer cells. *Am. J. Obstet. Gynecol.*, **166**, 1053–1061.

LIPPMAN M, BOLAN G AND HUFF K. (1976). The effects of oestrogens and antiestrogens on hormone-responsive human breast cancer in long-term tissue culture. *Cancer Res.*, **36**, 4595–4601.

LISITSYN N, LISITSYN N AND WILGER M. (1993). Cloning the differences between two complex genomes. *Science*, **259**, 946–951.

LUYTEN GP, HOOGEVEEN AT AND GALJAARD H. (1985). A fluorescence staining method for the demonstration and measurement of lysosomal enzyme activities in single cells. *J. Histochem. Cytochem.*, **33**, 965–968.

MACGREGOR GR AND CASKEY CT. (1989). Construction of plasmids that express *E. coli* beta-galactosidase in mammalian cells. *Nucleic Acids Res.*, **17**, 2365.

MULLICK A AND CHAMBON P. (1990). Characterization of the estrogen receptor in two antiestrogen-resistant cell lines, LY2 and T47D. *Cancer Res.*, **50**, 333–338.

MURPHY CS, MEISNER LF, WU SQ AND JORDAN VC. (1989). Short- and long-term estrogen deprivation of T47D human breast cancer cells in culture. *Eur. J. Cancer Clin. Oncol.*, **25**, 1777–1788.

MURPHY CS, PINK JJ AND JORDAN VC. (1990). Characterization of a receptor-negative, hormone-nonresponsive clone derived from a T47D human breast cancer cell line kept under estrogen-free conditions. *Cancer Res.*, **50**, 7285–7292.

NARDULLI AM AND KATZENELLENBOGEN BS. (1988). Progesterone receptor regulation in T47D human breast cancer cells: analysis by density labelling of progresterone receptor synthesis and degradation and their modulation by progestin. *Endocrinology*, **122**, 1532–1540.

OSBORNE CK, CORONADO-HEINSOHN EB, HILSENBECK SG, MCCUE BL, WAKELING AE, MCCLELLAND RA, MANNING DL AND NICHOLSON RI. (1995). Comparison of the effects of a pure steroidal antiestrogen with those of tamoxifen in a model of human breast cancer. *J. Natl Cancer Inst.*, **87**, 746–750.

PARIDAENS R. (1995). Hormonal resistance in breast cancer. In *Drug and Hormonal Resistance in Breast Cancer: Cellular and Molecular Mechanisms*. Dickson RB and Lippman ME. (eds) pp. 21-37. Ellis Horwood: Hemel Hempstead, UK.

PFEFFER U, FECAROTTA E AND VIDALI G. (1995). Coexpression of multiple estrogen receptor variant messenger RNAs in normal and neoplastic breast tissues and in MCF-7 cells. *Cancer Res.*, **55**, 2158-2165.

PINK JJ AND JORDAN VC. (1996). Models of estrogen receptor regulation by estrogens and antiestrogens in breast cancer cell lines. *Cancer Res.*, **56**, 2321-2330.

PINK JJ, JIANG SY, FRITSCH M AND JORDAN VC. (1995). An estrogen independent MCF-7 breast cancer cell line which contains a novel 80 kilodalton estrogen receptor related protein. *Cancer Res.*, **55**, 2583-2590.

PINK JJ, WU SQ, WOLF DM, BILIMORIA MM AND JORDAN VC. (1996). A novel 80 kilodalton estrogen receptor containing a duplication of exons 6 and 7. *Nucleic Acids Res.*, **24**, 962-969.

REESE CC, WARSHAW ML, MURAI JT AND SITTERI PK. (1988). Alternative models for estrogen and androgen regulation of human breast cancer cell (T47D) growth. *Ann. N.Y. Acad. Sci.*, **538**, 112-121.

ROBERSTON JFR. (1996). Oestrogen receptor: a stable phenotype in breast cancer. *Br. J. Cancer*, **73**, 5-12.

ROBINSON SP AND JORDAN VC. (1989). The paracrine stimulation of MCF-7 cells by MDA-MBA-231 cells: possible role in antiestrogen failure. *Eur. J. Cancer. Clin. Oncol.*, **25**, 493-497.

SARTORIUS CA, GROSHONG SD, MILLER LA, POWELL RL, RUNG L, TAKIMOTO GS AND HORWITZ KB. (1994). New T47D breast cancer cell lines for the independent study of progesterone B- and A-receptors: only antiprogestin-occupied B-receptors are switched to transcriptional agonists by cAMP. *Cancer Res.*, **54**, 3868-3877.

SOULE HD, VASQUEZ J, LONG A, ALBERT S AND BRENNAN M. (1973). A human cell line from a pleural effusion derived from a breast carcinoma. *J. Natl Cancer Inst.*, **51**, 1409-1416.

WANG Y AND MIKISICEK RJ. (1991). Identification of a dominant negative form of the human estrogen receptor. *Mol. Endocrinol.*, **5**, 1707-1715.

WELSHONS WV AND JORDAN VC. (1987). Adaptation of estrogen-dependent MCF-7 cells to low estrogen (phenol red-free) culture. *Eur. J. Cancer Clin. Oncol.*, **23**, 1935-1939.

WILDING G, LIPPMAN ME AND DICKSON RB. (1988). The cellular response of human breast cancer to estrogen. In *Progress in Clinial and Biological Research*, Hankins WD and Puett D (eds), **262**, pp. 181-196. Alan R Liss: New York.

WOLF DM AND JORDAN VC. (1994). The estrogen receptor from a tamoxifen stimulated MCF-7 tumor variant contains a point mutation in the ligand binding domain. *Breast Cancer Res. Treat.*, **31**, 129-138.