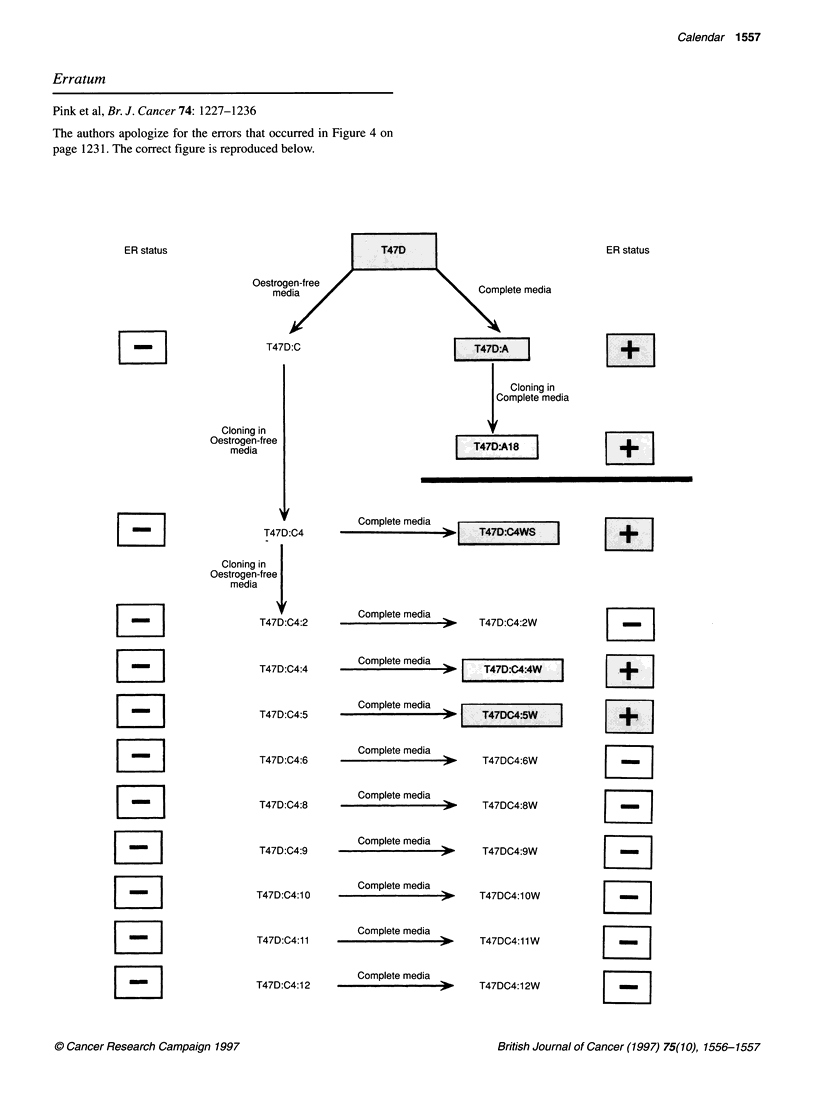# Erratum

**Published:** 1997

**Authors:** 


					
Calendar 1557

Erratum

Pink et al, Br. J. Cancer 74: 1227-1236

The authors apologize for the errors that occurred in Figure 4 on
page 1231. The correct figure is reproduced below.

ER status

ER status

Oestrogen-free

media

T47D:C

Cloning in

Oestrogen-free

media

T47D:C4

Cloning in

Oestrogen-free

media

T47D:C4:2

Complete media

: X ,

7 7 :7

7 z 7

7 7 _ n

Ef f E g Ei _  7 7

7  7g: 7  ::: D  t  7  7  7

E  f  7  E  f  E 0  .

S t .

=      T=<= s

Complete media                  -

_.: >     W 1  :ST4  ;: :w

_)m  1 7 70| j.

.'S.0t';~~~i St1S ;ttLP!f'''  -v9,X f.: 00S- I i

Complete media

>      T47D:C4:2W

T47D:C4:4          Complete media

Complete media
T47D:C4:4 : S f 49:454W:00::

Complete media

T47D:C4:6       -          --      >

Complete media
T47D:C4:8       -

Complete media
T47D:C4:9

Complete media

T47D:C4:12                         >

Complete media
T47D:C4:1 1

Complete media
T47D:C4:1 2

T47DC4:6W

T47DC4:8W
T47DC4:9W
T47DC4:1 OW
T47DC4:11 W
T47DC4:1 2W

British Journal of Cancer (1997) 75(10), 1556-1557

EEl

EEl
EEl
EEl
EEl
EEl
EEl
EEl
EEl
EEl

EEl

0 Cancer Research Campaign 1997